# Personality traits in chronic daily headache patients with and without psychiatric comorbidity: an observational study in a tertiary care headache center

**DOI:** 10.1186/1129-2377-14-22

**Published:** 2013-03-04

**Authors:** Marialuisa Rausa, Sabina Cevoli, Elisa Sancisi, Daniela Grimaldi, Gabriella Pollutri, Michela Casoria, Daniela Grieco, Alberto Bisi, Pietro Cortelli, Euro Pozzi, Giulia Pierangeli

**Affiliations:** 1 Department of Biomedical and Neuromotor Sciences-DIBINEM, University of Bologna – IRCCS Istituto delle Scienze Neurologiche di Bologna, Padiglione G, Ospedale Bellaria, via Altura 3, Bologna, 40139, Italy; 2 Department of Psychiatry, Bologna Local health Trust, Bologna, Italy

**Keywords:** Chronic daily headache, Medication overuse headache, Psychiatric comorbidity, MMPI-2

## Abstract

**Background:**

Previous studies suggest that patients with Chronic Daily Headache (CDH) have higher levels of anxiety and depressive disorders than patients with episodic migraine or tension-type headache. However, no study has considered the presence of psychiatric comorbidity in the analysis of personality traits. The aim of this study is to investigate the prevalence of psychiatric comorbidity and specific personality traits in CDH patients, exploring if specific personality traits are associated to headache itself or to the psychiatric comorbidity associated with headache.

**Methods:**

An observational, cross-sectional study. Ninety-four CDH patients with and without medication overuse were included in the study and assessed by clinical psychiatric interview and Mini International Neuropsychiatric Interview (M.I.N.I.) as diagnostic tools. Minnesota Multiphasic Personality Inventory-2 (MMPI-2), Hamilton Depression Rating Scale (HAM-D) were afterwards administered. Patients with and without psychiatric comorbidity were compared. Further analyses were made by splitting the whole group according to the headache diagnosis and the presence or not of medication overuse.

**Results:**

Psychiatric comorbidity was detected in 44 patients (46.8%) (group A) and was absent in the remaining 50 patients (53.2%) (group B). Mood and anxiety disorders were the most frequently diagnosed (43.6%).

In the overall group, mean scores of MMPI-2 showed a high level in the so-called neurotic triad; in particular the mean score in the Hypochondriasis subscale was in the pathologic area (73.55 ± 13.59), while Depression and Hysteria scores were moderate but not severe (62.53 and 61.61, respectively). In content scales, score in Health Concern was also high (66.73).

Group A presented higher scores compared to Group B in the following MMPI-2 subscales: Hypochondriasis (p = .036), Depression (p = .032), Hysteria (p < .0001), Hypomania (p = .030). Group B had a high score only in the Hypochondriasis subscale. No significant differences were found between chronic migraine (CM)-probable CM (pCM) plus probable medication overuse headache (pMOH) and chronic tension-type headache (CTTH)-probable CTTH (pCTTH) plus pMOH patients or between patients with and without drug overuse.

**Conclusions:**

The so-called “Neurotic Profile” reached clinical level only in CDH patients with psychiatric comorbidity while a high concern about their general health status was a common feature in all CDH patients.

## Background

Chronic daily headache (CDH) is not a universally recognized diagnosis but an umbrella term for a group of headache disorders occurring at least 15 days per month [1-3]. Unfortunately the classification and definition of CDH are still plagued with difficulties [4]. The CDH population comprises individuals with chronic tension-type headache (CTTH) and chronic migraine (CM), both of which may be associated with medication overuse [5-7]. The majority of patients reporting CDH have a history of episodic headache, mainly migraine without aura (MWOA), evolving into a chronic form over the years [8]. CDH is a major clinical concern and a common health risk, with a prevalence of approximately 3% to 5% in the adult population worldwide [9-11]. Identifying risk factors for progression has emerged as a major public health priority. Psychiatric comorbidity has been one of the risk factors most widely investigated for headache chronification due the significant role it may play in this process [12-16] and because it might be linked to medication overuse in migraineurs [17]. Many epidemiological and clinical studies have confirmed the elevated risk for mood and anxiety disorders in migraine and in CDH [18-23]. In particular, patients with CDH showed higher levels of anxiety and depressive disorders than patients with episodic migraine [19]. Some studies [19,24,25] hypothesized that patients with medication overuse headache (MOH) may differ psychologically from other headache patients because of a dependence-related behavior, but this hypothesis was not confirmed by more recent findings [26-28].

Personality traits assessed by the Minnesota Multiphasic Personality Inventory-2 (MMPI-2) [29] disclosed that patients with CDH showed the so-called “neurotic MMPI-2 profile” characterized by high scores in the first three scales: Hs (Hypochondriasis), D (Depression), and Hy (Hysteria) [30,31]. A recent study [27] comparing MMPI scores in MOH, episodic headache patients and healthy controls, showed that MOH and episodic headache patients displayed similar patterns, differentiating only in Hypochondriasis scale, and that there were no differences between the three groups in scales measuring dependence-related behavior. Another recent study [28] compared MOH sufferers and drug-addicted patients by means of MMPI-2 dependency scales showing that the two groups did not share personality characteristics linked to dependence. The authors argued that rather than a “true” addiction behavior, a different kind of “dependence” characterized headache patients related to the need to avoid pain. No study has hitherto explored if dependence behaviors in MOH patients are related to the psychiatric comorbidity often associated with CDH.

Aims of the present study were to: 1) investigate the prevalence of psychiatric comorbidity and specific personality traits in CDH patients, 2) investigate if specific personality traits characterize only patients with psychiatric comorbidity or were associated with the headache type.

## Methods

One hundred and five consecutive adult patients referred to the Headache Centre of the Department of Neurological Sciences of the University of Bologna and satisfying inclusion criteria for CDH (≥15 days/months for at least 3 months) with or without medication overuse, were recruited by expert neurologists (S.C., E.S., G.P.). Headache and drug overuse were classified according to the original [5] and the revised International Classification of Headache Disorders-II (ICHD-II) criteria [6]. Exclusion criteria were: age <18, secondary CDH assessed by clinical examination, biochemical tests or neuroimaging studies.

The study protocol included a psychiatric evaluation by means of a clinical assessment set up by expert psychiatrists (M.C., D.G., A.B.) and of the Mini International Neuropsychiatric Interview (M.I.N.I.) [32] in order to identify subjects with psychiatric comorbidity. Moreover the Hamilton Depression Rating Scale (HAM-D) [33] and the Minnesota Multiphasic Personality Inventory-2 (MMPI-2) [29,34] questionnaires were administered..

M.I.N.I. [32] is a short structured diagnostic interview for psychiatric disorders as defined by the Diagnostic and Statistical Manual of Mental Disorders, 4^th^ edition (DSM-IV) [35] and by the International Classification of Diseases, 10^th^ edition (ICD-10) [36]. It generates a positive diagnosis for the main Axis I DSM-IV disorders: mood, anxiety, eating and substance-related disorders. It also explores psychotic symptoms to exclude probable lifetime or current psychotic disorders.

HAM-D [33] is a depression test measuring the severity of clinical depression symptoms. It is a 21-item multiple choice questionnaire assessing depression in four levels: score under 7: no depression, score from 8 to 17: mild depression, score from 18 to 24: moderate depression, score over 25: severe depression.

MMPI-2 [29,34] is a 567 true-false item questionnaire composed of three validity and ten clinical scales. The questionnaire also includes content scales (clusters of items concerning the same psychological dimension and behavioral area) and supplementary scales which evaluate broad personality traits, generalized emotional distress and behavioral dyscontrol. For each scale, a T-score of 65 was considered as the level of clinical significance in the 95th percentile. MMPI-2 questionnaires were selected on the basis of the three validity scales.

M.I.N.I. and HAM-D were administered by a psychiatrist, while MMPI-2 was self-reported.

The institutional review board of the Department of Neurological Sciences of the University of Bologna approved the study protocol and all participants gave written informed consent. The study was performed in accordance with the ethical standards laid down in the 1964 Declaration of Helsinki.

### Data analysis

Descriptive statistics (means ± SD) were conducted on the sample features. The sample was divided into two groups according to M.I.N.I. results: patients with and without psychiatric comorbidity (Group A and Group B, respectively). Analysis of skewedness and kurtosis showed that data had a normal distribution. Further analyses were made by splitting the whole group according to the presence or absence of medication overuse, and according to the headache diagnosis: CM or probable CM (pCM) plus probable MOH (pMOH) *vs* CTTH or probable CTTH (pCTTH) plus pMOH according to the ICDH-II and MOH, CM and CTTH according to the ICDH-II revised criteria. Moreover we consider which preventive therapy patients were assuming.

Chi-squared and Student’s T-test were performed to compare data between groups. Data were analyzed using the statistical software SPSS 19.0 (Statistical Package for Social Science). Significance level was set at p < .05.

## Results

### Descriptive analysis

Ninety-four out of 105 patients with CDH and consecutively referred to the Headache Center were recruited. Eleven refused to participate in the study, 94 accepted to partecipate and signed an informed consent. All partecipating patients received a psychiatric evaluation by means of a clinical assessment and of the M.I.N.I. [32]. Seven patients withdrew their consent. To the 87 patients left, the HAM-D [33] and the MMPI-2 [29,34] questionnaires were administered.

According to the original ICHD-II criteria [5], four subjects (4.2%) had a diagnosis of CM, 18 ( 19.1%) of CTTH, 43 (45.7%) had a diagnosis of pCM plus pMOH, and 29 (30.9%) of pCTTH plus pMOH. According to ICHD-II revised criteria [6], four subjects (4.2%) had a diagnosis of CM, 18 (19.2%) of CTTH, and 72 (76.6%) of MOH.

Table 1 shows patients’ demographic and headache features. Drug overuse was significantly higher in pCM + pMOH patients compared to pCTTH + pMOH patients (43 vs 29, χ^2^ = 11.631; p < .001).

**Table 1:** Patients’ demographic and headache characteristics

**Gender**	
Male, n (%)	22 (23.4)
Female, n (%)	72 (76.6)
**Age** (yrs), mean ± sd (min-max)	48.51 ±14.31 (19-75)
**Educational level**	**n (%)**
Elementary/secondary school,	50 (53,2)
High or graduate school, n (%)	44 (44.7)
**Types of headache at onset**	**n (%)**
MWOA	78 (83.0)
MWOA + MWA	8 (8.5)
ETTH	5 (5.3)
CTTH	3 (3.2)
**Age of chronification** (yrs), mean ± sd	38.10 ±14.95
**Duration of chronification** (yrs), mean ± sd	10.21 ± 10.63
**Type of CDH**	**n (%)**
*(Revised ICHD-II diagnosis)*	MOH 72 (76.6)
	CTTH 18 (19.2)
	CM 4 (4.2)
**Type of CDH**	**n (%)**
*(ICDH-II diagnosis)*	CM 4 (4,2%)
	pCM, pMOH 43 (45,7%)
	CTTH 18 (19.2%)
	pCTTH, pMOH 29 (30.8%)

CTTH = chronic tension-type headache; ETTH = episodic tension-type headache; MWA = migraine with aura; MWOA = migraine without aura; MOH = medication overuse headache, CM = chronic migraine, pCM = probable CM, pCTTH = probable CTTH, pMOH = probable MOH.

### Psychiatric comorbidity

Descriptive analysis of M.I.N.I. showed that 44 patients (46.8%) presented psychiatric comorbidity (group A), whereas 50 patients (53.2%) did not present any psychiatric comorbidity (group B) (Table 2). In particular, within group A, 20 patients (21.2%) were classified as mood disorder sufferers, 16 patients (17%) had an anxiety disorder, six patients (6.4%) had anxiety and mood disorder and two patients (2.1%) had other psychiatric disorders (eating disorders).

**Table 2:** Psychiatric comorbidity

	***Group A***				***Group B***	***Total***
	**Mood disorder**	**Anxiety disorder**	**Mood and anxiety disorder**	**Other psychological disorder**		
CM	1 (1.1%)	0 (0%)	0 (0%)	0 (0%)	3 (2.1%)	4 (4.2%)
pCM plus pMOH	8 (8.5%)	7 (7.4%)	2 (2.1%)	1 (1.1%)	25 (26.6%)	43 (45.7%)
CTTH	4 (4.2%)	1 (1.1%)	3 (3.1%)	0 (0%)	10 (10.6%)	18 (19.1%)
pCTTH plus pMOH	7 (7.4%)	8 (8.5%)	1 (1.1%)	1 (1.1%)	12 (12.8%)	29 (30.8%)
**Total**	**20 (20.2%)**	**16 (17.0%)**	**6 (6.4%)**	**2 (2.1%)**	**50 (53.2%)**	**94 (100%)**

CM = Chronic migraine, pCM = probable chronic migraine, pMOH = probable medication overuse headache, CTTH = chronic tension-type headache, pCTTH = probable chronic tension-type headache (according to the original ICHD-II criteria). Group A = patients with psychiatric comorbidity Group B = patients without psychiatric comorbidity.

No significant differences in psychiatric comorbidity frequency were found between CM or pCM plus pMOH and CTTH or pCTTH plus pMOH patients (p = .219), or between patients with and without drug overuse (p = .534). No significant differences were found in the percentage of Group A and Group B patients assuming antidepressant or mood stabilizer as preventive therapies (10/44 Group A and 16/50 Group B, χ^2^ = 0.596; p = .440).

### MMPI-2

Among the 94 patients, only 87 correctly completed the questionnaire, and 4 questionnaires were excluded because they did not achieve validity’s level described in the MMPI-2 manual. Statistical analyses were made in the 10 clinical scales. In the overall group, mean scores of MMPI-2 showed a high level in the so-called neurotic triad. In particular the mean score in the Hypochondriasis subscale was in the pathologic area (73.55 ± 13.59), while Depression and Hysteria scores were moderate but not severe (62, 53 and 61.61, respectively). Secondary analyses were made on content and supplementary MMPI-2 scales. In content scales, score in Health Concern was also high (66.73).

Group A showed significantly higher scores in the following MMPI-2 subscales when compared to Group B (Table 3): Hypochondriasis (p = .031), Depression (p = .008), Hysteria (p < .0001), Psychopatic Deviate (p = .025), Psychasthenia (p = .0,19), Schizophrenia p = .017), Hypomania (p = .025). Group B had a high score only in the Hypochondriasis subscale (70.38 ± 12.78).

**Table 3:** **Mean and standard deviation (*Mean*** **±** *sd) *of MMPI-2 scores*

		***Total N = 74***	***Group A N = 39***	***Group B N = 35***	**P**
MMPI	Hs (Hypochondriasis)	**73.55 **(13.60)	**76.80 **(13.79)	**70.38 **(12.78)	*0.03
Clinical Scales	D (Depression)	62.53 (12.61)	**66.19 **(12.49)	58,95 (11.81)	*0.00
	Hy (Hysteria)	61.61 (13.36)	**66.78 **(12.93)	56.57 (11.87)	*0.00
	Pd (Psychopathic Deviate)	53.85 (10.27)	56.39 (9,08)	51.38 (10.85)	*0.02
	Mf (Masculinity-feminility)	46.99 (9.85)	47.76 (8.48)	46.23 (11.09)	*0.49
	Pa (Paranoia)	55.49 (10.82)	56.63 (9.56)	54.38 (11.93)	*0.35
	Pt (Psychasthenia)	57.47 (10.78)	60.27 (10.61)	56.74 (10.34)	*0.02
	Sc (Schizophrenia)	55.75 (10.11)	58.41 (9.98)	53.14 (9.65)	*0.02
	Ma (Hypomania)	46.62 (11.36)	49.44 (10.87)	43.88 (11.29)	*0.02
	Si (Social introversion)	57.79 (10.94)	59.90 (10.56)	55.74 (11.03)	*0.08

*T-scores > 65 (considered to be the level of clinical significance) are in bold. Group A = patients with psychiatric comorbidity; Group B = patients without psychiatric comorbidity. Hyp + Dep + Hys = ”Neurotic profile”. * p < .05.

In the content scales, group A had high score in Health Concern (Group A = 69,24, Group B = 64,29; p > .05). The two groups did not show any high score in content scales, but differentiated in: Anxiety (Group A = 61,95, Group B = 57,40; p = .047), Depression (Group A = 60,76, Group B = 55,50; p = .025), Family Problems (Group A = 59,29, Group B = 54,40; p = .046); Work Interference (Group A = 58,56, Group B = 52,67; p = .021).

In the supplementary scales no high scores were found, but the two groups also differentiated in: Anxiety (Group A = 62,51, Group B = 56,60; p = .026) College Maladjustment (Group A = 64,95, Group B = 59,45; p = .026), Gender Feminine (Group A = 50,12, Group B = 44,57; p = .009), Post-Traumatic Stress disorder (Group A = 60,14, Group B = 54,88; p = .022); Addiction Potential Scale (Group A = 49.02, Group B = 43,38; p = .008).

No significant differences were found between CM or pCM plus pMOH and CTTH or pCTTH plus pMOH patients or between patients with and without drug overuse (Table 4).

**Table 4:** **Mean and standard deviation (*Mean ± sd) *of MMPI-2 scores in CM-pCM + pMOH versus CTTH-p CTTH + pMOH and MOH versus NO MOH**

	***CM and pCM + pMOH***	***CTTH and pCTTH + pMOH***	**p**	**MOH**	**NO MOH**	**p**
	***N = 42***	***N = 45***		***N = 66***	***N = 21***	
Hs (Hypochondriasis)	**75.21 **(11.66)	**71.35** (14.82)	0.17	**73.96 **(13.07)	**70.85 **(14.64)	0.39
D (Depression)	63.90 (9.88)	60.37 (14.56)	0.18	63.57 (11.48)	57.38 (14.88)	0.09
Hy (Hysteria)	62.85 (10.28)	59.66 (15.39)	0.26	62.22 (12.97)	58.00 (13.70)	0.22
Pd (Psychopathic Deviate)	54.90 (9.82)	52.11 (9.82)	0.22	54.36 (9.82)	50.61 (12.26)	0.21
Mf (Masculinity-feminility)	47.90 (9.69)	47.04 (10.31)	0.69	47.68 (9.52)	46.76 (11.48)	0.74
Pa (Paranoia)	56.69 (9.93)	54.40 (12.12)	0.34	56.53 (10.35)	52.28 (12.97)	0.18
Pt (Psychasthenia)	58.02 (10.63)	56.13 (10.92)	0.42	57.04 (10.68)	57.04 (11.25)	0.99
Sc (Schizophrenia)	55.38 (10.05)	55.53 (10.36)	0.94	55.72 (10.15)	54.61 (10.35)	0.67
Ma (Hypomania)	47.92 (10.20)	45.60 (12.12)	0.33	46.75 (10.60)	46.61 (13.33)	0.96
Si (Social introversion)	57.76 (10.20)	57.51 (11.34)	0.91	58.22 (9.67)	55.76 (13.70)	0.45

T-scores > 65 (considered to be the level of clinical significance) are in bold. CM = Chronic migraine, pCM = probable chronic migraine, pMOH = probable medication overuse headache, CTTH = chronic tension-type headache, pCTTH = probable chronic tension-type headache (according to the original ICHD-II criteria).

### HAM-D

We analyzed 87 valid questionnaires. The mean value of the total sample at Hamilton Depression Scale was 6.4 and it remained in the normative range indicative for absence of depression. Group A (n = 43) had a scale mean value indicative for the presence of mild depression (9.47 ± 4.26), significantly higher than group B (n = 42; 3.48 ± 3.24) (t = 7.37, p < .0001). No significant differences were found between CM or pCM plus pMOH and CTTH or pCTTH plus pMOH patients (p = .345) or between patients with and without drug overuse (p = .994).

## Discussion

Our study evaluated the prevalence of psychiatric comorbidity and specific personality traits in CDH patients. Headache and drug overuse were classified using both the original [5] and the revised International Classification of Headache Disorders-II (ICHD-II) [6] criteria to clearly differentiate the type of prevalent headache (migraine *vs* tension-type headache) and the presence of drug overuse.

In our sample drug overuse is significantly increased in pCM + pMOH patients with respect to pCTTH + pMOH patients, probably due to the pain severity of their attacks.

Of the 94 subjects included in the sample, 46.8% showed psychiatric comorbidity. The disorders most frequently diagnosed were mood and anxiety disorders (43.6%). These data are in line with some previous literature [26,37-39], indicating that the most common psychiatric conditions related to migraine were depression, bipolar disorders, anxiety [19] and somatoform disorders. However, the relationship between CDH and psychiatric disorders is still matter of debate in the literature, this being also due to the different headache diagnostic criteria and the different methods adopted in most of the studies to assess psychiatric disorders [12-16]. Verri and colleagues [21] found an association between CDH and at least one psychiatric disorder in 90% of their patients. Juang and colleagues [22] found that the frequency of any type of anxiety disorder was significantly higher in patients with CM than in those with CTTH. This was not confirmed in our sample in which no differences were found in psychiatric comorbidity between the migraine and the tension-type headache groups, and between patients with and without drug overuse. These findings are in agreement with the results obtained by Atasoy and colleagues [40] who analyzed psychiatric comorbidity in 89 MOH patients and did not find any difference in MOH patients with pre-existing episodic tension-type headache (ETTH) with respect to those with pre-existing MWOA.

Psychiatric comorbidity has often been clinically discussed rather than systematically studied. The use of M.I.N.I., a structured clinical psychiatric interview [41], helped us to establish a diagnosis on reliable and valid diagnostic criteria. Although M.I.N.I. is considered a somewhat overinclusive instrument in ‘making diagnosis’ [42], it indicated the presence of depression in our sample, while HAM-D results revealed that the mood disorder was milder than expected. Moreover, after clinical interviews, psychiatrists reported that psychiatric disorders in MOH appear in subthreshold forms rather than as full-blown disorders. Even if the percentage of patients assuming prophylactic therapies with antidepressant or mood stabilizer drugs was similar in both groups, with and without psychiatric comorbidity, we can not exclude a role of these drugs in the final results.

Previous MMPI-2 results stressed the presence of the “neurotic MMPI-2 profile” in headache patients, a profile characterized by a high level of depression, hypochondria and hysteria [27,30,31]. We found that in our sample, the neurotic triad was detected only in patients with psychiatric comorbidity, while patients without psychiatric comorbidity displayed a high score only in the Hypochondriasis subscale, indicating high concern for their health status. The presence of the “neurotic MMPI-2 profile” in chronic headache has already been the focus of previous studies [27,28] arguing that these personality traits appeared to be a reaction to chronic pain rather than a specific feature of headache patients [43]. Our results suggest for the first time that the “neurotic MMPI-2 profile” was not associated to headache per se, but was a dominant feature in headache patients who showed comorbidity with psychiatric disorders. However the cross-sectional design of the study does not permit to reveal any casual relationship.

The two groups also differentiated for scores in Addiction Potential Scale: patients with psychiatric comorbidity had higher scores than patients without, even if the scores fell in the normal range. Furthermore, the presence of medication overuse was not a discriminating factor. These data support Galli et al.’s [28] hypothesis that medication overuse in CDH patients might not be related to dependence, but rather might be a consequence of chronic pain. From this perspective, medication overuse could be seen as the only way patients know to cope with their pain and maintain a normal lifestyle. However, this hypothesis conflicts with other studies on dependence in patients with MOH [25]. Specific instruments are needed to assess dependence behavior in MOH patients as it seems to have different theoretical constructs from classic dependence behavior [27].

A high score in MMPI-2 Health Concern subscale was found in all CDH patients suggesting concern for their general health condition, and not only for their headache. This result is concordant with the elevated score also found in the Hypochondria clinical scale.

We acknowledge the limitations of the present study, the principal one being that it was performed in a tertiary care headache clinic so that the investigated sample may not represent the whole spectrum of CDH patients in the general population. Another limitation was that MMPI-2 was properly completed only by 83 of the 94 subjects included. Another major problem was the diagnostic distinction of CDH and drug overuse that remains controversial and could introduce a selection bias. We tried however to overcome this limitation, which is intrinsic to all studies investigating headache chronification, by applying the IHS revised criteria for headache diagnosis.

## Conclusion

Our study showed that the so-called Neurotic Profile (high level of hypochondria, depression and hysteria) reached a clinical level only in patients with psychiatric comorbidity and that all CDH patients, independently from psychiatric comorbidity, had a high score in hypochondria evaluation suggesting a high concern for their general health status. Large population-based studies are needed to confirm this original finding and future prospective studies might also evaluate how hypocondria is a risk factor for transformation from episodic to chronic headache and if it is possible to modify this aspect by applying cognitive behavioral interventions.

## Abbreviations

CDH: Chronic daily headache; CTTH: Chronic tension-type headache; pCTTH: Probable chronic tension-type headache; CM: Chronic migraine; pCM: Probable chronic migraine; MWOA: Migraine without aura; MWA: Migraine with aura; ETTH: Episodic tension-type headache; MOH: Medication overuse headache; pMOH: Probable medication overuse headache; MMPI-2: Minnesota Multiphasic Personality Inventory; ICHD-II: International classification of headache disorders-II; M.I.N.I: International neuropsychiatric interview; HAM-D: Hamilton depression rating scale

## Competing interests

The authors have no conflicts of interest in connection with the submitted article.

## Authors’ contributions

MR performed the statistical analysis, was involved in the interpretation of data and was primarily involved in drafting the manuscript. SC made substantial contribution to conception and design of the study, to acquisition and interpretation of data, to critical revision of the manuscript for important intellectual content. ES made substantial contribution the design, in the coordination of the study and in acquisition and analysis of data. DG have been involved in drafting the manuscript and revising it critically. GP made substantial contribution in acquisition of data. MC made substantial contribution in acquisition of data. DG made substantial contribution in acquisition and analysis of data. AB made substantial contribution in acquisition of data. PC made substantial contribution to conception and design of the study. He was involved in the interpretation of data, critical revision of the manuscript for important intellectual content. EP made substantial contribution to conception, design of the study and to interpretation of data. GP contribute to conception of the study and to the acquisition of data. GP made substantial contribution in drafting the manuscript, revising it critically and given final approval of the version to be published. All authors read and approved the final manuscript.
